# Predictive Modeling of Localized Mobile App to Improve Snakebite Management in Ghana

**DOI:** 10.1371/journal.pntd.0014435

**Published:** 2026-06-08

**Authors:** Eric Nyarko, Aashna Uppal, Nicholas Amani Hamman, Louis Agyekum, Pascal Antwi, Nuhu Mohammed, Obu-Amoah Ampomah, Iddrisu Abugbil Atubiga, Trudie Lang

**Affiliations:** 1 Department of Statistics and Actuarial Science, College of Basic and Applied Sciences, University of Ghana, Legon, Accra, Ghana; 2 The Global Health Network, Nuffield Department of Medicine, University of Oxford, Oxford, England, United Kingdom; 3 Snakebite Treatment and Research Hospital, Kaltungo, Gombe, Nigeria; 4 Department of Economics, University of Ottawa, Ottawa, Canada; 5 Department of Statistics, Western Michigan University, Kalamazoo, Michigan, United States of America; Fundação de Medicina Tropical Doutor Heitor Vieira Dourado: Fundacao de Medicina Tropical Doutor Heitor Vieira Dourado, BRAZIL

## Abstract

**Background:**

Snakebite envenoming (SBE) is a significant yet often overlooked public health crisis that primarily affects impoverished communities. Mobile applications (apps) can be effective tools for managing snakebites. To meet the World Health Organization’s (WHO) goal of reducing SBE by 50% by 2030, app developers must consider regional users’ preferences to ensure their apps provide relevant and accurate information. This study predicted the importance of various functionalities of a mobile app for managing snakebites based on user preferences. Our objective was to provide quantitative evidence on which functions should be prioritized to inform the development of a customized local app to enhance snakebite care in Ghana.

**Methods:**

A cross-sectional survey using a quantitative statistical experiment design method was conducted to identify healthcare workers’ preferences for vital mobile app functions for managing snakebites. Participants were selected from two deprived and predominantly rural districts in the Eastern region of Ghana through a multi-stage sampling technique. The attributes used in the questionnaire were developed based on literature reviews and focus group discussions, and a statistical block design was employed to create the choice tasks. To rigorously evaluate the performance of the machine learning (ML) models and reduce the risk of overfitting, we employed 5-fold cross-validation and multiple evaluation metrics. The data were analyzed using seven ML models.

**Results:**

The four most vital mobile app functions identified by the participants were “step-by-step assistance for victims and first responders” (utility estimates (β) =0.3924; 95% confidence interval (CI): 0.3183 to 0.4665), “providing educational and training materials” (β = 0.2243; 95% CI: 0.1500 to 0.2987), “identifying venomous snakes through clinical evidence or symptoms” (β = 0.1718; 95% CI: 0.0982 to 0.2454) and “identifying venomous snake biodiversity in your area/region” (β = 0.0898; 95% CI: 0.0176 to 0.1620). Conversely, the app functions that were less favored included “platform for sharing snakebite treatment experiences” (β = -0.2503; 95% CI: -0.3268 to -0.1738) and “designing educational games about snakes” (β = -0.3995; 95% CI: -0.4737 to -0.3252). These findings align with subgroup analyses by gender, suggesting a consistent understanding of needs across different demographic groups.

**Conclusions:**

This study provides quantitative evidence on which mobile app functions should be prioritized to inform the development of a customized local app to improve management and care for snakebite victims in Ghana and other sub-Saharan African countries.

## Author summary

Snakebite envenoming (SBE) is a significant yet often overlooked public health crisis that primarily affects impoverished communities. Mobile applications can serve as effective tools for managing snakebites. To meet the World Health Organization’s (WHO) goal of reducing SBE by 50% by 2030, app developers must consider regional users’ preferences to ensure their apps provide relevant and accurate information. This study used a quantitative best-worst scaling design and machine learning models to predict which functionalities of a mobile app for managing snakebites are most important, based on the lived experience of healthcare workers involved in snakebite management in two deprived and predominantly rural districts in the Eastern region of Ghana. Four key functions were identified as most important: “step-by-step assistance for victims and first responders,” “providing educational and training materials,” “identifying venomous snakes through clinical evidence or symptoms,” and “recognizing the biodiversity of venomous snakes in your area.” Conversely, the features considered least important were “a platform for sharing snakebite treatment experiences” and “designing educational games about snakes.” These results not only highlight the preferred functionalities of a potential snakebite management app but also indicate alignment with subgroup analyses by gender, suggesting a consistent understanding of needs across different demographic groups. Our findings provide quantitative evidence to guide the development of a customized local app to improve snakebite care. By leveraging digital health solutions, we can improve health outcomes and support the WHO’s goal of halving SBE by 2030.

## Introduction

Snakebite envenoming (SBE) is a significant yet often overlooked public health crisis, primarily affecting impoverished communities, with minimal advocacy on their behalf [1]. Despite the extensive impact of snakebites worldwide, reliable data on morbidity and mortality are limited [2]. It is estimated that there are approximately 5.4 million snakebites and 1.8 to 2.7 million cases of envenomation globally each year, resulting in around 81,000–138,000 deaths and approximately 400,000 cases of permanent disabilities, such as blindness, tissue loss, amputations, and post-traumatic stress disorder [3]. In Africa, between 435,000 and 580,000 snakebites require treatment annually [4].

Medically important snakes found in Ghana include the saw-scaled viper (*Echis ocellatus*), the puff adder (*Bitis arietans*), the black-necked spitting cobra (*Naja nigricollis*), and the Western Green Mamba (*Dendroaspis viridis*) [5]. However, the incidence and mortality rates of snakebites vary by region. A community-level study in northern Ghana found an annual snakebite incidence of 56.4 cases per 100,000 inhabitants, with a mortality rate of 1.35 per 100,000 inhabitants [6]. In contrast, data from health facilities in the Volta region indicated an incidence rate of 15.8 snakebite cases per 100,000 population and a case fatality rate of 0.4% [7]. Given the situation surrounding SBE, effective prevention and treatment strategies are crucial.

As part of a global initiative to reduce SBE mortality and disability by 50% by 2030, the World Health Organization (WHO) established a strategic roadmap in 2019 [8]. This roadmap focuses on strengthening health systems, empowering communities, ensuring safe and effective treatment, and enhancing partnerships and resources. With advances in technology, digital tools will play a vital role in implementing the WHO roadmap by improving data collection and health systems [9,10]. These tools facilitate better resource allocation, empower communities with improved access to health information, and promote patient engagement. Digital solutions also support telemedicine and remote monitoring, enhancing the quality of care and outreach while fostering collaboration among stakeholders, which is essential for the effective execution of the WHO strategy.

While digital health interventions, such as mobile applications (apps) for snake identification, telemedicine platforms for remote consultations, and artificial intelligence-driven diagnostic tools, offer transformative potential in snakebite management [9,11], they are not without limitations. For instance, some app functions may be user-unfriendly or less accessible [12]. Of the 16 mobile-based health apps currently available for snakebite management worldwide, most lack regional customization for sub-Saharan African (SSA) snake species and are designed explicitly for regions such as India (11 apps), the United States (1 app), the United Kingdom (1 app), Sri Lanka (1 app), and South Africa (2 apps), neglecting other high-risk regions in SSA [9]. The African Snakebite Institute Snakes app (from South Africa) is the only free app focused on South African snake species, but its limited utility in neighboring countries limits its usefulness. Another significant challenge is the difficulty users face when selecting the most suitable app, as most are designed for Android devices (i.e., India 11 apps), with few compatible with both Android and iOS (i.e., eSnakes Southern Africa–by mydigitalearth.com) [9]. Additionally, many of these apps require internet connectivity to function. Navigating apps can be complicated because each one has its own interface and features, which can lead to user confusion and frustration [13]. Meanwhile, perceived ease of use of digital tools significantly increased adoption rates [14].

Despite their limitations, mobile apps can be a valuable tool for snakebite management. They provide a platform for health authorities to communicate with the public, offer access to medical care, and promote education on snakebite prevention and control [15]. To meet the WHO’s 2030 targets to reduce the burden of snakebite envenoming, app development must prioritize underrepresented regions, such as SSA, especially Ghana, where SBE is prevalent, through partnerships with local health systems and communities. Due to limited resources and recognizing that technology has its limitations, no single app can provide all necessary functions. A trade-off is required to prioritize the most essential features. This approach ensures that mobile apps provide relevant, accurate information that effectively addresses local needs, ultimately empowering both healthcare providers and communities.

There is currently a gap in research on quantifying the importance of mobile app functions from a local user’s perspective and by gender subgroups. Addressing this gap could yield valuable insights for future successful local app development. As telecommunications continue to advance [16,17], mobile apps have the potential to play an increasingly vital role in managing health [10,18,19], including snakebites [12,20]. To ensure the effectiveness of these apps, involving local healthcare workers in their design and development is essential to avoid user confusion and frustration. Their perspectives are crucial, as they are on the front lines of treating and managing snakebite patients. The lived experiences and insights of these healthcare workers are invaluable for developing relevant interventions, such as mobile apps for snake identification, telemedicine platforms for remote consultations, and AI-driven diagnostic tools for snakebite management. This study aimed to predict the importance of a mobile app’s functions for managing snakebites based on user preferences. It focuses on healthcare workers managing snakebite patients in two deprived and predominantly rural districts in the Eastern region of Ghana, namely Kwahu Afram Plains North (KAPN) [21] and Kwahu Afram Plains South (KAPS) [22], aiming to inform the development of a tailored app to improve local snakebite care. The hypothesis guiding this study was that healthcare workers share the same preferences for the mobile app functions for managing snakebites.

## Materials and methods

### Ethics

The study was granted ethical approval by the Ghana Health Service Ethics Review Committee (GHS-ERC073/04/24) and followed all ethical guidelines and regulations. All participants were given a clear explanation of the study’s purpose, and written informed consent was obtained. They were informed that participation was voluntary and that they could choose whether to take part in the study without consequences.

### Best-worst scaling method

Best-worst scaling (BWS) is a quantitative survey method that typically asks respondents to identify their most and least preferred choices [23], under real/hypothetical conditions, regarding a health program characterized by specific attributes. Participants are presented with various combinations of these attributes across different choice sets, each containing at least three options. A significant advantage of BWS is that it allows respondents to weigh competing characteristics against each other, rather than simply ranking or rating them [24,25]. This approach provides researchers with deeper insights into the relative importance of each attribute. The advantages of BWS, particularly when compared to discrete choice experiments [26], have been noted in a prior study [27]. Results from BWS can effectively inform policymakers about public preferences, which can then be integrated into health program development, ultimately guiding more effective health policies. The use of BWS in health research is widespread [24] and valuable for understanding the factors that influence public preferences for new health initiatives before they are implemented [13,28].

### Selection of attributes

The development of the BWS questionnaire was guided by recommended best practices, qualitative research, and reviews of existing evidence to inform attribute selection and design. This process consisted of some key steps. Initially, we created a pool of 18 potential attributes by conducting an online search for features of mobile applications (apps) for snakebite management. This step established a foundational understanding of current mobile app usage in snakebite management. In the second step, we conducted a focus group discussion with a panel of local experts comprising 20 healthcare workers, a public health researcher, and a mobile app developer. The purpose of this discussion was to review the attributes and identify potential issues to enable necessary revisions. The panel provided valuable input, including revisions and the addition of new information, such as ensuring the app offers details on the availability and cost of WHO-approved antivenoms at healthcare facilities and pharmacies. Their feedback helped ensure that the attributes were clear, concise, and easy to understand. Ultimately, we confirmed 11 plausible attributes (functions) for use in developing the BWS questionnaire. The finalized attributes included: Snakebite treatment protocol (app provides intuitive, scientific clinical protocols to follow when dealing with snakebite cases); Step-by-step assistance to victims and first responders (provides clear, actionable clinical and biological advice on steps to take in case of snakebite); Provides education and training (app provides a range of useful education and training materials and links about snake bite envenoming, first aid prevention of snakebite, and snake biology); Identify venomous snake biodiversity in your area/region; Design snake games to teach about snakes in your area/region (provides habitat-specific quizzes); Identify venomous snakes using clinical evidence/symptoms presented by a snakebite victim (neurological, muscular, cardiac, renal effects); Identify venomous snakes in your lab using morphological clues (enlarged teeth; venom fangs generally grooved at the front or with a venom duct orifice near the tip with +/- at the back; there are always normal teeth in front of these venom fangs); Works offline (app designed to run all functions without an active internet connection); Surveillance and monitoring of snakebite victims; Platform for snakebite treatment experience sharing; Information on the availability and cost of WHO-approved antivenoms in healthcare facilities and medicine/pharmacy sales outlets.

### Statistical experiment design

To ensure the research design’s effectiveness, we employed a block experimental design with 11 choice scenarios, each replicated twice. Each scenario included five mobile app functions for snakebite management. This method guarantees an equal probability of selecting each attribute in the questionnaire. Since no prior information was available on the parameters, we constructed the design assuming equal choices for each option. Overall, each attribute occurred 5 times across all sets, with each occurring only once in each choice scenario generated from the block design. In this study, each participant was required to make a total of 44 choices (22 for the best option and 22 for the worst).

### Study site, participant sampling and recruitment

It is worthwhile mentioning that this study was part of a larger cross-sectional survey conducted from August to December 2024 in two deprived and predominantly rural districts in the Eastern region of Ghana, namely Kwahu Afram Plains North and Kwahu Afram Plains South. These districts are located within the savannah vegetation zone, which includes both the savannah transitional zone and savannah woodland [21,22]. These areas have reported incidents of snakebites [29,30]. Common snakes found in such zones include those with venoms that cause hemorrhagic effects, primarily *Echis ocellatus* [5]. The clinical manifestations of bites from this snake can lead to renal complications due to coagulopathy, alongside marked local effects such as pain, severe swelling, bruising, blistering, and moderate to severe necrosis. Additionally, *Naja nigricollis* is known for its neurotoxic effects. Bites from this snake can result in local pain, swelling, blistering, tissue destruction, bleeding, venom-induced coagulopathy, and spontaneous systemic hemorrhaging (in areas such as the gums, gastrointestinal tract, and brain). Thus, healthcare professionals must understand clinical presentations to accurately identify the cause of envenomation and manage the case effectively. Moreover, approximately three-quarters of the communities are located on islands within water bodies, including Volta Lake, the River Afram, and the Obosom River, which limits access to higher-level referral care and leads to delayed referral pathways [31]. Most residents in other settlement communities walk between 25 and 35 minutes to reach the main district hospital in Donkorkrom [31]. Additionally, about 59.9% of the population seeks healthcare outside of their immediate settlement. While information on smartphone ownership among healthcare workers in these districts is scarce, mobile phone ownership among the general population stands at 22.9% in KAPN [21] and 27.1% in KAPS [22]. Since healthcare workers are generally more educated and better connected professionally, it is likely that smartphone access among them is higher. In rural areas of Ghana, smartphones are the most owned functional information communication technology device, with 58.9% of individuals aged 12 years and older using them [16].

**Fig 1:**
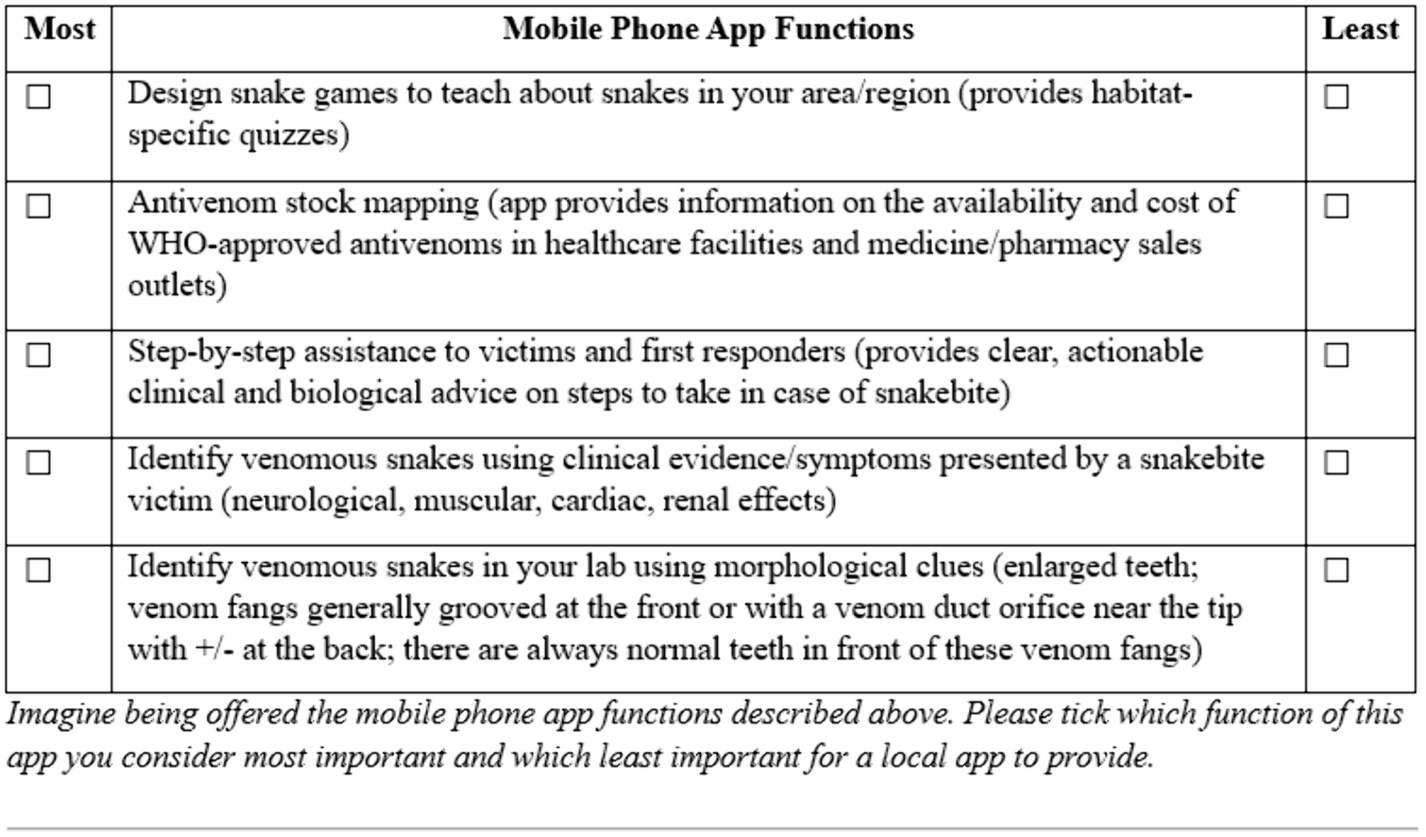
An example choice scenario.

The minimum number of respondents required for this study was 70, based on a standard sample size calculation [32] and the available choice scenarios. A 20% upward adjustment was made to account for the expected non-response rate, which added 14 respondents to the total. Consequently, the adjusted sample size for the study was set at 84. However, to enhance the statistical power of our results, a higher sample size was preferable for this cross-sectional study. Therefore, we oversampled and recruited a total of 137 healthcare workers involved in the treatment and management of snakebite patients in the two districts, as similarly reported in our previous study [33]. We employed a multistage sampling technique to select three communities: Donkorkrom, Tease, and Amankwakrom, which served as clusters for our sample [33,34]. From these clusters, we randomly selected 137 healthcare professionals engaged in treating and managing snakebite patients across three health facilities within the communities. The healthcare workers were approached during their working hours at their designated facilities to complete the questionnaires. During data collection, respondents were presented with choice scenarios one at a time. Participants were asked based on their lived experience to select two attributes/functions: one they considered most important and the other they considered least important for the app to provide. They recorded their responses on paper and with a pen. Enumerators ensured neutrality by not observing responses in real time and maintained participants’ autonomy through peer-based data collection. An example choice scenario is presented in Fig 1.

### Data management and analysis

Data collection included real-time validation checks to ensure a complete dataset with no missing data [33]. Enumerators conducted initial checks for incomplete responses in the survey tools and encouraged respondents to finish any unfinished sections without observing their responses in real time. No specific issues were identified during the internal consistency checks that could affect our results. To rigorously evaluate ML models performance and reduce the risk of overfitting, we employed a 5-fold cross-validation strategy, which improved model performance compared to the hold-back validation approach. The full dataset was randomly divided into five mutually exclusive folds. In each of the five iterations, four folds were combined to train the model, while the remaining fold was used as a validation set to compute performance metrics. This process was repeated so that each fold served as the validation set once, ensuring that all data contributed to both training and validation and yielding a more stable estimate of model generalizability. In this study, the predictor variables related to mobile app functions used to manage snakebite were treated as generic attributes, while the response variable was effects coded and treated as continuous. Data were analyzed using seven ML models, including Support Vector Machines (SVM), Fit Least Squares, Least Absolute Shrinkage and Selection Operator (LASSO), Ridge Regression, Elastic Net, and Generalized Regression (Forward and Pruned Forward Selection). Model performance was evaluated using the Mean Root Average Squared Error (MRASE), calculated as the average of the Root Average Squared Error across the cross-validation folds, with lower values indicating superior prediction. Additionally, computation time, defined as the time required to fit the model to the training data, was measured to assess model efficiency, particularly for potential real-time or large-scale applications. The corrected Akaike Information Criterion (AICc), and the Bayesian Information Criterion (BIC) are also reported. All statistical analyses for this study were performed using JMP Pro statistical software. The approach employed in the current study, although primarily based on ML models, could not be viewed as solely empirical or entirely dependent on black-box algorithms. By incorporating established methods from the literature, the black box is illuminated, providing insights into the contribution and importance of each attribute in the models [35].

## Results

### Participant characteristics

This study involved 137 healthcare workers, comprising 83 females (60.6%) and 54 males (39.4%). The participants represented a diverse array of professional backgrounds and areas of expertise, as similarly reported in our previous research [33,34]. Within this participant group, 53 females (63.9%) and 45 males (83.3%) were aged 31 years or older, as reported in Table 1. Importantly, 37.3% of the female participants reported having 3–5 years of experience specifically in managing and treating snakebite patients. This experience level suggests a valuable familiarity with the complexities involved in such cases. The majority of respondents were either certified, enrolled, or general nurses, with 73 females (88.0%) and 23 males (42.6%) falling into these categories. This underscores the critical role that nursing professionals play in the healthcare landscape. Additionally, among male respondents, 12 individuals (22.2%) held positions as physician assistants, clinical officers, or medical doctors, illustrating the range of healthcare roles represented in this study.

**Table 1:** Characteristics of the study population by gender.

Variable	Category	Female (n = 83)	Male (n = 54)
**Age (years)**	18-30	30 (36.1%)	9 (16.7%)
	31+	53 (63.9%)	45 (83.3%)
**Occupation**	Certified, Enrolled, or General Nurses	73 (88.0%)	23 (42.6%)
	Community Health Nurses	4 (4.8%)	9 (16.7%)
	Pharmacists or Dispensing Technicians	2 (2.4%)	10 (18.5%)
	Physician Assistants, Clinical Officers, and Medical Doctors	4 (4.8%)	12 (22.2%)
**Experience (years)**	1-2	25 (30.1%)	7 (13.0%)
	3-5	31 (37.3%)	14 (25.9%)
	6+	27 (32.5%)	33 (61.1%)

### Model performance evaluation metrics

A comparative evaluation of the seven ML models revealed that the Ridge Regression model achieved the lowest MRASE (0.59698), indicating the highest accuracy in predicting the held-out utility scores. The LASSO and Elastic Net models performed similarly well (MRASE = 0.59700). In contrast, the SVR model displayed the highest prediction error (MRASE = 0.62504). In terms of computational efficiency, the Generalized Regression models, specifically Pruned Forward Selection and Forward Selection, were the fastest, with times of 172 milliseconds (ms) and 191 ms, respectively. On the other hand, SVR was the most computationally intensive, taking 9757 ms. Ridge Regression proved to be the most effective models, providing the best balance between predictive accuracy and computational efficiency, with a processing time of 2159 ms. As a result, Ridge Regression was selected as the top-performing model. This model also demonstrated excellent goodness-of-fit, achieving the lowest AICc value of 2787.6966 and the lowest BIC value of 2851.3023. In contrast, the standard/traditional maximum difference model had a significantly higher AICc value of 8240.2924 and a BIC value of 8293.3039. We chose the Ridge Regression model because it offers the best balance between predictive performance, computational efficiency, and overall fit.

### Ridge regression model results

The Ridge Regression model was used to assess the relative importance of each mobile app function. The standardized regression coefficients from the final Ridge Regression model were interpreted as preference weights, which were then transformed into utility scores. These utility scores indicate the relative importance of each mobile app function. The results of the full Ridge Regression model regarding the app’s functionalities are provided in Table 2 with an insight based on gender subgroups. The model is statistically significant (p = 0.0001), highlighting the differences in healthcare workers’ preferences for various mobile app functions. Each attribute’s importance was measured relative to the app’s “works offline” function, which served as the reference point for interpretation. However, the functions “identify venomous snakes in your lab using morphological clues,” “snakebite treatment protocol,” “surveillance and monitoring of snakebite victims,” and “antivenom stock mapping” were not statistically significant. This indicates that healthcare workers did not demonstrate a strong preference for these functions compared to the reference function. The analysis indicated that “step-by-step assistance to victims and first responders” was the most important and favored app function among participants, with utility estimate (β) of 0.3924 (95% CI: 0.3183 to 0.4665; p < 0.0001), followed by “provides education and training materials,” (β = 0.2243; 95% CI: 0.1500 to 0.2987; p < 0.0001). This emphasizes the need for practical assistance and educational resources in snakebite scenarios. The attributes “identify venomous snakes using clinical evidence/symptoms” and “identify venomous snake biodiversity in your area/region” were statistically significant and of intermediate importance, reflecting a desire for both diagnostic support and heightened awareness of local snake species. In contrast, “platform for snakebite treatment experience sharing” (β = -0.2503; 95% CI: -0.3268 to -0.1738; p < 0.0001) and “design snake games to teach about snakes” (β = -0.3995; 95% CI: -0.4737 to -0.3252; p < 0.0001) were the least favored or deprioritized features, suggesting a lesser interest in gamified learning or shared experiences in the context of snakebite treatment.

**Table 2:** Ridge regression model results for each function of the mobile phone app for managing snakebite envenoming.

Function of mobile phone app	Utility estimate (β)	SE	Wald ChiSquare	Prob > ChiSquare	Lower 95%	Upper 95%
Design snake games to teach about snakes in your area/region (provides habitat-specific quizzes)	-0.3995	0.0378	111.2761	<0.0001*	-0.4737	-0.3252
Identify venomous snake biodiversity in your area/region	0.0898	0.0368	5.9469	0.0147*	0.0176	0.1620
Identify venomous snakes in your lab using morphological clues (enlarged teeth; venom fangs generally grooved at the front or with a venom duct orifice near the tip with +/- at the back; there are always normal teeth in front of these venom fangs)	-0.0010	0.0408	0.0006	0.9801	-0.0810	0.0789
Identify venomous snakes using clinical evidence/symptoms presented by a snakebite victim (neurological, muscular, cardiac, renal effects)	0.1718	0.0375	20.9173	<0.0001*	0.0982	0.2454
A platform for snakebite treatment experience sharing	-0.2503	0.0390	41.1166	<0.0001*	-0.3268	-0.1738
Provides education and training (app provides a range of useful education and training materials and links about snake bite envenoming, first aid prevention of snakebite, and snake biology)	0.2243	0.0379	34.9948	<0.0001*	0.1500	0.2987
Snakebite treatment protocol (app provides intuitive, scientific clinical protocols to follow when dealing with snakebite cases)	0.0454	0.0364	1.5565	0.2122	-0.0259	0.1168
Step-by-step assistance to victims and first responders (provides clear, actionable clinical and biological advice on steps to take in case of snakebite)	0.3924	0.0377	107.8331	<0.0001*	0.3183	0.4665
Surveillance and monitoring of snakebite victims	-0.0634	0.03845	2.7183	0.0992	-0.1387	0.0119
Antivenom stock mapping (app provides information on the availability and cost of WHO-approved antivenoms in healthcare facilities and medicine/pharmacy sales outlets	0.0259	0.0353	0.5417	0.4617	-0.0432	0.0951
Works offline (app designed to run all functions without an active internet connection)	Reference level					
**Model Fit Statistics**						
AICc	2787.6966					
BIC	2851.3023					
MRASE	0.59698					
Negative Log-Likelihood	1358.8487					
P-value	0.0001					

*Statistical significant apps; MRASE, mean root average squared error; SE, standard error; AICc, corrected Akaike information criteria; BIC, Bayesian information criteria.

Interestingly, the subgroup analyses by gender largely aligns with the full model results, though the attribute “identify venomous snake biodiversity in your area/region” is not statistically significant for either male or female participants. “Step-by-step assistance for victims and first responders” is also the most preferred app feature for both genders, with β = 0.4311 (95% CI: 0.3367 to 0.5255; p < 0.0001) for females and β = 0.2928 (95% CI: 0.1734 to 0.4121; p < 0.0001) for males. Following this, “provides education and training materials” had β = 0.2355 (95% CI: 0.1370 to 0.3339; p < 0.0001) for females and β = 0.1820 (95% CI: 0.0688 to 0.2952; p = 0.0016) for males. Conversely, “platform for snakebite treatment experience sharing” and “design snake games to teach about snakes” were significant but were less favored by both gender groups. Additionally, “surveillance and monitoring of snakebite victims” showed significance for females with β = -0.1230 (95% CI: -0.2212 to -0.0248; p = 0.0140).

The differences in each mobile app function compared to the overall average are presented in Table 3. A positive mean difference indicates that a specific function is significantly more preferred than the overall average. Conversely, a negative mean difference suggests that the function is less preferred than the overall average. Among the mobile app functions, those relating to “step-by-step assistance for victims and first responders,” “providing education and training materials,” and “identifying venomous snakes using clinical evidence or symptoms” showed notably higher mean values. In contrast, functions such as “a platform for sharing snakebite treatment experiences,” “designing snake games to educate about snakes,” and “surveillance and monitoring of snakebite victims” exhibited lower mean values. Overall, these findings indicate that healthcare workers prioritized practical, clinically relevant functions that directly enhance emergency response and patient management. Meanwhile, features focused on educational gaming, experience sharing, and surveillance were considered less critical for immediate snakebite care.

**Table 3:** Differences of each function of mobile phone app from the overall average.

Function of mobile phone app	Functions of mobile phone app	Difference	SE	Adjusted Lower 95%	Adjusted Upper 95%	t Ratio	Adjusted Prob > |t|
Design snake games to teach about snakes in your area/region (provides habitat-specific quizzes)	Avg	-0.4275	0.0241	-0.4957	-0.3593	-17.72	<.0001*
Identify venomous snake biodiversity in your area/region	Avg	0.0618	0.0224	-0.0017	0.1253	2.75	0.0629
Identify venomous snakes in your lab using morphological clues (enlarged teeth; venom fangs generally grooved at the front or with a venom duct orifice near the tip with +/- at the back; there are always normal teeth in front of these venom fangs)	Avg	-0.0290	0.0285	-0.1097	0.0516	-1.02	0.9776
Identify venomous snakes using clinical evidence/symptoms presented by a snakebite victim (neurological, muscular, cardiac, renal effects)	Avg	0.1438	0.0236	0.0769	0.2106	6.08	<.0001*
A platform for snakebite treatment experience sharing	Avg	-0.2783	0.0259	-0.3516	-0.2050	-10.74	<.0001*
Provides education and training (app provides a range of useful education and training materials and links about snake bite envenoming, first aid prevention of snakebite, and snake biology)	Avg	0.1963	0.0241	0.1279	0.2647	8.11	<.0001*
Snakebite treatment protocol (app provides intuitive, scientific clinical protocols to follow when dealing with snakebite cases)	Avg	0.0174	0.0217	-0.0441	0.0790	0.80	0.9965
Step-by-step assistance to victims and first responders (provides clear, actionable clinical and biological advice on steps to take in case of snakebite)	Avg	0.3644	0.0239	0.2965	0.4322	15.20	<.0001*
Surveillance and monitoring of snakebite victims	Avg	-0.0914	0.0250	-0.1621	-0.0206	-3.66	0.0028*
Antivenom stock mapping (app provides information on the availability and cost of WHO-approved antivenoms in healthcare facilities and medicine/pharmacy sales outlets	Avg	-0.0020	0.0198	-0.058	0.0540	-0.10	1.0000
Works offline (app designed to run all functions without an active internet connection)	Avg	-0.0280	0.0271	-0.1048	0.0488	-1.03	0.9754

*Statistical significant apps; Avg, overall average; SE, standard error.

## Discussion

This study, to the authors’ knowledge, is the first to use the quantitative BWS method alongside ML models to offer policymakers insights into the importance of various mobile app functions for managing snakebites. The research primarily focused on the lived experiences of healthcare workers involved in snakebite management in two deprived and predominantly rural districts in the Eastern region of Ghana, as they are at the forefront of diagnosing, treating, and caring for snakebite patients. Their insights are essential for guiding the development of a localized app to improve snakebite care in Ghana. Our research findings do not support the hypothesis that healthcare workers share the same preferences for mobile app functions used to manage snakebites.

It has been recognized that the severity of SBE is largely influenced by the quality of first aid or management provided to the victim before they reach a hospital [15,36]. In countries like Australia, which implement effective field management strategies such as the pressure-immobilization technique and have widely available effective antivenoms, the incidence of snakebite fatalities is significantly lower than in countries lacking these preventive and therapeutic measures [37]. In this study, we found that the most important function of an app is “step-by-step assistance for victims and first responders.” This finding is noteworthy because it suggests that healthcare professionals prioritize a localized app that provides immediate, detailed guidance to snakebite victims, first responders, and bystanders. Such an app should address common harmful practices such as tourniquet use and traditional remedies while including recommendations for WHO approved first aid actions, such as immobilizing the affected limb, reassuring and calming the victim, and removing constrictive items (like rings, anklets, and bracelets) from around the bite site, before quickly transporting the individual to a healthcare facility or contacting emergency services [38,39]. This preference underscores health professionals’ belief that providing clear, actionable steps can serve as a robust strategy to reduce snakebite-related morbidity and mortality.

In this study, participants emphasized the importance of an app function that “provides educational and training materials”. This highlights the need for resources on snakebite management to empower local communities [39] and enhance healthcare workers’ knowledge, skills, and practices [40,41]. It is essential to develop a localized mobile app that provides access to snakebite information, promotes health education, and offers training materials for caregivers, religious leaders, community elders, school teachers and students, and healthcare workers, including medical doctors, nurses, and pharmacists. These training and educational materials should be aligned with guidelines issued by the Ministry of Health and the Ghana Health Service, as well as WHO-validated recommendations for preventing and managing SBE. An example is the WHO Africa Regional Office (AFRO) guidelines for the prevention and clinical management of snakebites in Africa [42]. These guidelines are utilized in the training and ongoing educational programs for physicians and nurses [41].

Another vital function of the app studied was the ability to “identify venomous snakes through clinical evidence or symptoms.” It is important to develop a localized mobile app that helps users identify the snake’s species or genus by analyzing specific signs and symptoms. This is essential for guiding treatment decisions and ensuring proper management of snakebite victims, particularly in rural health facilities where diagnostic tools for determining the snake species responsible for the bite may not be available [43]. Understanding snake characteristics and the specific clinical manifestations is essential for species identification and effective management of SBE [5]. Snake venoms can cause different types of effects: neurotoxic, which affects the nervous system and can lead to respiratory paralysis (as seen in snakes like cobras, kraits, and mambas), hemotoxic, which impairs the circulatory system and can result in coagulopathy, hemorrhage, and tissue necrosis (common in the Viperidae family), and predominantly cytotoxic (characteristic of spitting cobras) [44,45].

Our study also emphasized that a crucial feature of the app should be the ability to “identify venomous snake biodiversity in your area/region.” Therefore, the app should incorporate profiles of snake species that are specific to each district or region, based on the ecological zones and epidemiological patterns in Ghana. However, knowledge of the distribution of snake species across Ghana’s agro-ecological zones remains limited [5]. Understanding these distribution patterns can provide important epidemiological information to help identify snake bites and the types of venom involved, which is essential for improving diagnosis and ensuring timely, appropriate treatment of snakebite envenomation [46]. There is a need for expert herpetologists [43] to identify medically important snake species across Ghana’s agro-ecological zones to inform the app’s customization. A comprehensive understanding of venomous snake biodiversity in these areas can help identify the most relevant species, thereby informing the establishment of treatment and safety protocols [46–48]. This knowledge can also inform healthcare strategies such as antivenom distribution, conservation efforts, open up research opportunities, and support effective habitat management.

Although app functions such as “a platform for sharing snakebite treatment experiences,” “designing educational games about snakes,” and “surveillance and monitoring of snakebite victims” remain important, their trade-off or lower overall preference indicates that they may not be highly prioritized, but they nonetheless require attention to improve snakebite management and care. While all these functions contribute to snakebite management and care, the varying levels of preference indicate where improvements and customization might be necessary to better meet user needs and preferences in this critical area.

While limited statistical power may lead to results that do not show significant findings, it was not surprising that the snakebite treatment protocol, intended to provide intuitive, scientifically based guidelines for managing snakebite cases, was found to be non-significant in this study, as a previous study has reported low adherence to snakebite treatment protocols in some districts in Ghana [49]. Therefore, the development of a localized mobile app that integrates protocols aligned with current national and WHO-recommended procedures is crucial for guiding treatment decisions and ensuring effective management of snakebite victims. The absence of recommended guidelines for treating snakebites in health facilities contributes to poor treatment outcomes [50].

This study has some limitations. While our findings may apply to other regions of Ghana and SSA due to the probability sampling technique used, we focused only on two deprived and predominantly rural districts and healthcare workers due to financial constraints. This narrow focus could make our findings more representative of these professionals’ needs, including the possibility of social desirability bias in their responses. Future studies should include perspectives from diverse groups, such as community members, traditional healers, policymakers, and researchers. Features such as multilingual support, geographic distribution maps, photo-based snake identification, emergency/rescue contacts, myth-busting content, and real-time expert guidance were omitted. This omission may introduce bias into our findings. The high number of choice scenarios in this study may lead to respondent fatigue/cognitive burden. Although this study focused on forced-choice approach, it might artificially enhance the perceived utility of the app’s functions. Future studies should include an opt-out option. Because we employed a cross-sectional design, we did not assess how subjects’ preferences for mobile app functions might change over time. Lastly, the study relied on self-reported data from healthcare workers, and the validity of these reports could not be verified.

In conclusion, this study employed quantitative BWS method and ML models to predict the importance of various functions in a mobile app for managing snakebites, based on user preferences. The four most important functions identified by participants were: “step-by-step assistance for victims and first responders,” “providing educational and training materials,” “identifying venomous snakes through clinical evidence or symptoms,” and “recognizing the biodiversity of venomous snakes in your area.” In contrast, the features deemed least important were “a platform for sharing snakebite treatment experiences” and “designing educational games about snakes.” These findings also indicate alignment with subgroup analyses by gender, suggesting a consistent understanding of needs across different demographic groups. Our findings provide quantitative evidence to guide the prioritization of mobile app functions for developing a customized local app to improve snakebite care. By leveraging digital health solutions, we can improve health outcomes and support the WHO’s goal of halving SBE by 2030. The implementation of the app within the health system or ecological zones will prioritize strong security measures to build trust and confidence among potential users. This will include minimizing or anonymizing personally identifiable information to protect patient confidentiality, encrypting data transmission, and requiring password-protected user authentication. Additionally, there will be adherence to ethical standards for digital health in accordance with Ghana’s health data governance frameworks and the Ghana Health Service’s digital health strategy.
